# Alterations of BCCIP, a BRCA2 interacting protein, in astrocytomas

**DOI:** 10.1186/1471-2407-9-268

**Published:** 2009-08-04

**Authors:** Jingmei Liu, Huimei Lu, Hiroko Ohgaki, Adrian Merlo, Zhiyuan Shen

**Affiliations:** 1 Department of Radiation Oncology, The Cancer Institute of New Jersey, UMDNJ-Robert Wood Johnson Medical School, 195 Little Albany St, New Brunswick, NJ 08903, USA; 2 Pathology Group, International Agency for Research on Cancer, World Health Organization, 150 Cours Albert Thomas, 69372 Lyon Cedex 08, France; 3 Neurosurgery and Laboratory of Molecular Neuro-Oncology, Departments of Surgery and Research, University Hospitals, Spitalstrasse 21, CH-4031 Basel, Switzerland

## Abstract

**Background:**

Loss of heterozygosity of chromosome 10q26 has been shown to be associated with the aggressiveness of astrocytic tumors (or astrocytomas), but the responsible gene(s) residing in this region has not been fully identified. The *BCCIP *gene is located at chromosome 10q26. It encodes a BRCA2 and CDKN1A (p21) interacting protein. Previous studies have shown that down-regulation of BCCIP impairs recombinational DNA repair, G1/S cell cycle checkpoint, p53 trans-activation activity, cytokinesis, and chromosome stability, suggesting a potential role of *BCCIP *in cancer etiology. In this study, we investigated whether *BCCIP *is altered in astrocytomas.

**Methods:**

Genomic DNA from 45 cases of grade IV astrocytic tumor (glioblastoma) tissues and 12 cases of normal tissues were analyzed by quantitative PCR. The BCCIP protein expression in 96 cases of grade II–IV astrocytic tumors was detected by immunohistochemistry (IHC). IHC staining of glial fibrillary acid protein (GFAP), a marker for astrocytic cells, was used to identify cells of the astrocytic lineage.

**Results:**

We found that BCCIP protein is expressed in normal cells with positive staining of GFAP. However, BCCIP protein expression was not detectable in ~45% of all astrocytic tumors, and in > 60% in the grade IV glioblastoma. About 45% glioblastoma have significant (p < 0.01) reduction of *BCCIP *gene copy number when compared to normal DNA. Furthermore, the frequency of lacking BCCIP expression is associated with the aggressiveness of astrocytic tumors.

**Conclusion:**

Our data implicate a role of BCCIP in astrocytic tumorigenesis, and lack of *BCCIP *may be used as a marker for astrocytomas.

## Background

Astrocytic tumor, or astrocytoma, is one of the most lethal forms of brain cancer although they rarely metastasize. Improvement of astrocytoma interventions requires further understanding on the genetic alterations associated with these tumors. Loss of heterozygosity (LOH) of chromosome 10q (especially region 10q25.3–26.2) is frequently associated with astrocytoma [1-11]. LOH on chromosome 10q26 is highly correlated with a poor prognosis for astrocytoma [1-7,9-12], and perhaps is the one of the most reliable prognosis markers associated with a worse outcome [8,9,13]. Thus, identifying the gene(s) in this region associated with this form of brain tumor is of critical importance for astrocytoma intervention. In addition to brain tumor, LOH of 10q26 has been shown to be associated with prostate cancers, endometrial cancers, and lung cancers [14-17], further suggesting the importance of identifying the tumor related gene(s) on 10q26.

Genomic instability is a major driving force for tumor progression. The *BRCA2 *gene plays critical roles in the maintenance of genomic stability by regulating homologous recombination, and *BRCA2 *defects are associated with predisposition to various human cancer. Although mutation of *BRCA2 *itself is involved in only a small population of human cancer, the germline BRCA2 mutations are highly penetrative to cancer. This suggests that the entire molecular pathway of BRCA2 are critical for cancer prevention, and other proteins related to BRCA2 may contribute to additional tumors [18]. Thus identifications and analyses of novel BRCA2 interacting protein may provide unique opportunities to identify additional genetic factors involved in tumorigenesis. We and others have previously reported BCCIP as a BRCA2 and CDKN1A [19] interacting protein [20-23]. We have shown that the chromatin bound fraction of BCCIP co-localizes with BRCA2 and contributes to BRCA2 and RAD51 nuclear focus formation [22]. A ~50% down-regulation of BCCIP is sufficient to inhibit homologous recombination, and both the BRCA2 and p21 interaction domains of BCCIP play a role in regulating homologous recombination [22,24,25]. Furthermore, BCCIP has been shown to play a role in supporting p53 transcription activity, and the completion of cytokinesis during mitosis [26,27]. All these support a critical role of BCCIP in the maintenance of genomic stability.

The human *BCCIP *gene is located at 10q26.1 [20], and a recent report suggested that BCCIP may be absent in at least one astrocytoma cell line [28]. We previously showed that the A172 astrocytic brain tumor cell line has reduced expression of BCCIPα [20]. Given that *BCCIP *is involved in important processes relevant to the maintenance of genome stability and its localization at 10q26, we investigated the possibility of BCCIP alterations in more than 100 cases of astrocytomas. Because brain tumor in the astrocyte lineage are the most common type [29,30], we emphasized our analyses on the astrocytic tumors including the grade IV glioblastoma multiforme. We found that BCCIP is down-regulated in a major portion of these brain tumors. Furthermore, BCCIP down-regulation is correlated with the aggressiveness of astrocytomas. These data suggest BCCIP alteration as a new marker for astrocytomas.

## Methods

### Quantitative Real-Time PCR detection of BCCIP copy number

A total of 45 cases of anonymous genomic DNA were isolated from grade IV astrocytoma as previously reported [5,31]. Twelve cases of DNA from peripheral blood leukocytes were used as control for normal BCCIP gene dosage determination. Real-time PCR was done using the SYBR Green strategy with a DNA Engine Opticon™ 2 Real-Time Detection System (M J Research Inc. South San Francisco, CA). Each pair of primers was optimized to amplify only the anticipated PCR products, which were verified by DNA sequencing and melting curve analysis. The primers for BCCIP are as follows: exon 5: 5'-TGC TTT CTA GGG TAC CCA GTG-3' (forward) and 5'-CCA CAG GCT TGG TGG TGT C-3'(reverse, 129 bp); exon 6: 5'-GGC ACA CAG AAC CAA TAA GCC-3' (forward) and 5'-CAT TTG CAA ACA TTA ACG CAG C-3' (reverse, 136 bp); exon 7: 5'-TCA ACT ACT CAG TGC AGG AGG AG-3' (forward) and 5'-CAG TTT ATC CAT GAT TTC GTT CAT C-3' (reverse, 135 bp); exon 9: 5'-AAG TGA CAG CCC TGG TTT CTC-3' (forward) and 5'-ATG AGC CTC CTA AAT CCC TGA C-3' (reverse, 130 bp). To normalize the copy number of BCCIP gene, the copy number of glyseraldehyde-3-phosphate dehydrogenase (GAPDH) was used as an internal control. The primers for GAPDH are: 5'-AAC GTG TCA GTG GTG GAC CTG-3' (forward) and 5'-AGT GGG TGT CGC TGT TGA AGT-3' (reverse, 160 bp), as validated and described previously [5,32]. This pair of primers amplifies a single band as confirmed by melting curve analysis with the DNA Engine Opticon™ 2 Real-Time Detection System (M J Research Inc. South San Francisco, CA), and confirmed to be the coding gene of GAPDH by DNA sequencing. To our knowledge, this pair of primer does not amply GAPDH pseudogenes. In each PCR analyses, we used 25 μl of reaction volume containing 4 μl of DNA, 100 μM each of the primers, and 12.5 μl of 2× master mix from DyNAmo HS SYBR Green qPCR kit (Finnzymes). Standard curve to calibrate DNA concentration was performed for all amplifications for each pair of primers. The PCR reaction tubes were first incubated for 15 minutes at 95°C, followed by 40 cycles of 30 seconds at 95°C, 20 seconds at optimized annealing temperature depending on the primers, and 20 seconds at 72°C.

### Antibodies and Immunohistochemistry of BCCIP and GFAP in brain tumors

Rabbit anti-BCCIPα/β antibodies were reported previously [20]. Paraffin embedded brain tumor tissue array slides (Catalog No GL801, BS17001, and BS17014) were purchased from US Biomax (Rockvile, MD). The core diameter is 1.5 mm, and the slides have thickness of 5 μm. The pathological grade of tumor on the array were provided by the slide supplier, and later confirmed by us when the slides were evaluated. The astrocytoma grade II–IV in pathology diagnosis is equivalent to low-grade moderately-differentiated astrocytoma, anaplastic astrocytoma, and undifferentiated glioblastoma multiforme respectively. These slides originally contain more than 100 cases of independent astrocytoma, 12 cases of oligodendroglioma, and 5 cases of ependymomas. Some of the cases were eliminated from data analysis due to lack of tumor tissues on the cores of the particular cases. This was caused by tissue detachment from the slides during the staining process, or because the presence of tumor tissue cannot be verified. Thus, a total 96 cases of astrocytomas, 6 cases of oligodendrogliomas, and 5 cases of ependymomas are reported here. Because objective of this study is to perform a retrospective determination on whether BCCIP is altered in existing specimens without contacting with living individual, no information on the patient identities and clinical outcomes are available for the investigators, approval for human subject research was deemed unnecessary.

The astrocytomas are in the astrocytic lineage, and the astrocytic cells often express a unique marker glial fibrillary acid protein (GFAP). Based on staining of non-tumor brain tissue, BCCIP is not uniformly expressed in brain tissue, but its expression can be detected in GFAP positive cells (see Results section). Therefore, we stained GFAP to identify the astrocytic tumor cells in the sections. Double staining of the same slide with GFAP and BCCIP turned out to be challenging with the available antibodies. Thus, two neighboring serial sections of these slides were stained with anti-GFAP monoclonal antibody (Chemicon International, Temecula, CA) and anti-BCCIP antibody respectively using routine immunohistochemistry (IHC) protocols. Briefly, slides were de-paraffinized with xylene for 10 min, and repeated three times. The antigen was retrieved in citrate acid buffer (pH6.5) by steaming in a rice cooker for 20 min. Following antigen retrieval, the endogenous peroxidase activity was blocked at room temperature by 20 minutes of incubation with 1% H_2_O_2 _in methanol. The slides were blocked with 5% milk in TBS-T (25 mM Tris-HCl pH 7.5, 150 mM NaCl, 0.1% Tween20) for 30 min at room temperature, and then incubated with 1:100 diluted affinity-purified rabbit polyclonal anti-BCCIP antibodies for 3 hrs at room temperature. This BCCIP antibody has been reported previously [20].

Following 3 times wash with TBS-T, the slides were incubated for 1 hr at room temperature with anti-Rabbit IgG secondary antibody (1:100) that was conjugated with horseradish peroxidase. The chromogenic substrate diaminobenzidine (DAB) was used as a chromogen to stain BCCIP positive cells (brown color). Haematoxylin was used as contrast staining for BCCIP negative cell nuclei (blue color). After mounting, the slides were observed under microscope. When the BCCIP nuclear stain was found in less than 5% of tumor cells, the case was scored as BCCIP negative. When more than 5% tumor cells are stained BCCIP positive, the case was considered BCCIP positive. For GFAP staining and evaluation, the same as BCCIP was used, except anti-GFAP monoclonal antibody was used as first antibody, and anti- mouse IgG (1:100) was used as the secondary antibody which was conjugated with horseradish peroxidase.

## Results

### Reduction of BCCIP gene dosage in brain tumors

**Figure 1:**
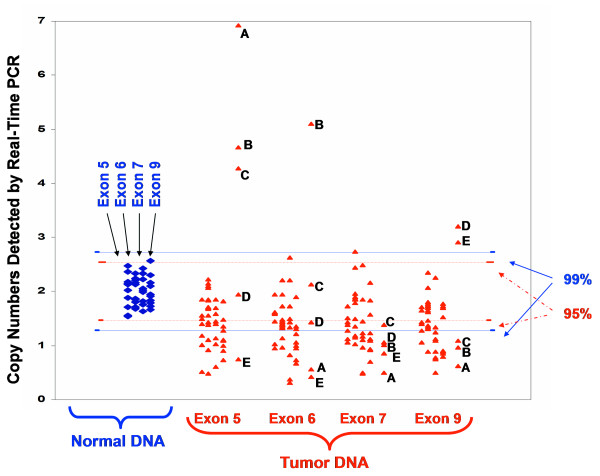
**Copy numbers of BCCIP exons detected by quantitative Real-Time PCR**. The copy number of exons 5, 6, 7, and 9 were measured in 10 cases of normal brain tissue DNA (diamond marker), and 45 cases of glioblastoma DNA (see Materials and Methods for details). The 95% and 99% confidence ranges of the copy number in normal tissues are marked. Many tumor DNAs showed reduction of BCCIP copy numbers. However, five cases of tumor DNA (marked as cases A-E) have reduced BCCIP copy numbers at some exons but increased in others. These re-arrangements likely cause inactivation of the BCCIP gene.

The BCCIP gene is located at chromosome 10q26.2 [20], a region frequently altered in brain tumors. The *BCCIP *gene also functions in cell cycle control, homologous recombination, mitosis, and is required to maintain the transactivation activity of wild type p53 [19,22-24,26,27]. Therefore, we were interested in determining whether altered BCCIP is associated with brain tumors. We first estimated the *BCCIP *gene copy numbers in genomic DNA from 45 cases of glioblastomas (grade IV). Due to the limited amount DNA available for this study, we used quantitative PCR to assess the BCCIP gene dosage. We performed Real-Time PCR using four independent sets of primers within exons 5, 6, 7, and 9 of *BCCIP *according to the genomic structure reported previously [33]. First, the relative level of BCCIP gene was determined from DNA of lymphocytes (see Methods). This provides a spectrum of BCCIP signals in normal tissues. The average of these signals from the normal DNA, and its standard deviation were calculated. Based on the Gaussian (or normal) distribution, the 99% confidence limit (p < 0.01) was set at 2.58 fold of the standard deviation, and the 95% confidence limit (P < 0.05) was set at 1.96 fold of the standard deviation. Then, the relative dosage of BCCIP gene in tumor DNA was compared with the distribution of normal DNA. If the copy numbers of two independent BCCIP exons are less than the 99% confidence line of the normal DNA, a "*BCCIP *loss" was scored for this case of tumor. We found that 20 out of 45 cases of glioblastoma DNA have significant loss of *BCCIP *with p < 0.01, an overall rate of 44.5%. In addition, 14 cases (31.1%) have *BCCIP *loss with 0.01 < p < 0.05. Thus a total of ~75% glioblastomas have BCCIP loss (p < 0.05). It was worth-noting that five cases of brain tumors (labeled as case A, B, C, D, and E in Figure 1) have reduced copy numbers in some exons, but higher copy numbers in other parts of the gene, indicating that other types of alteration such as regional amplification of the gene could have occurred in addition to deletion. Southern blot analyses for these tumor DNAs were impossible due to lack of sufficient DNA specimens. Nevertheless, our data indicate that *BCCIP *gene loss or alteration is common in glioblastomas.

### Loss of BCCIP protein expression in brain tumor

**Figure 2:**
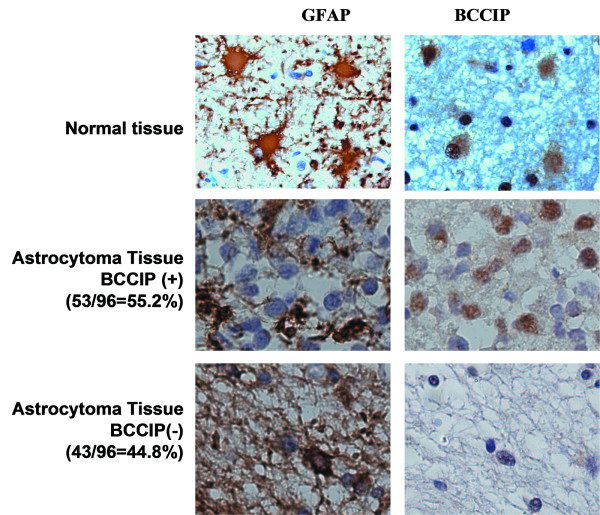
**Lack of BCCIP expression in brain tumors**. Serial tissue sections were stained for GFAP and BCCIP separately. Both GFAP and BCCIP are stained in brown color. Hematoxylin (blue) was used to counter stain the nuclei. Shown are example brain tissues stained with antibodies against GFAP and BCCIP (magnification is 40 × 10). Top panel is a representative non-tumor section. The middle panel is an example section of BCCIP positive tumor, and the bottom panel shows a BCCIP negative tumor section.

To confirm the loss of BCCIP, we measured the BCCIP protein expression in brain tumor tissue sections. These cases were not related to the cases presented in Figure 1, but were obtained from an independent source (US Biomax, Rockvile, MD) in the form of tissue arrays, because no tissue slides were available for the same cases as reported in Figure 1. The pathological grades of these tumors were provided by the supplier of the tissue arrays, but confirmed by experienced pathologist co-authors (HL and HO) based on the sections. Because majority of the tumor on the slides are astrocytoma, and most astrocytic tumor cells express the unique marker glial fibrillary acid protein (GFAP) [34,35], we first checked if BCCIP is expressed in normal GFAP positive cells. Using serial sections to stain normal brain tissue with anti-GFAP and anti-BCCIP antibodies (Figure 2, top panels), we found that BCCIP protein is mainly expressed in GFAP positive cells.

**Figure 3:**
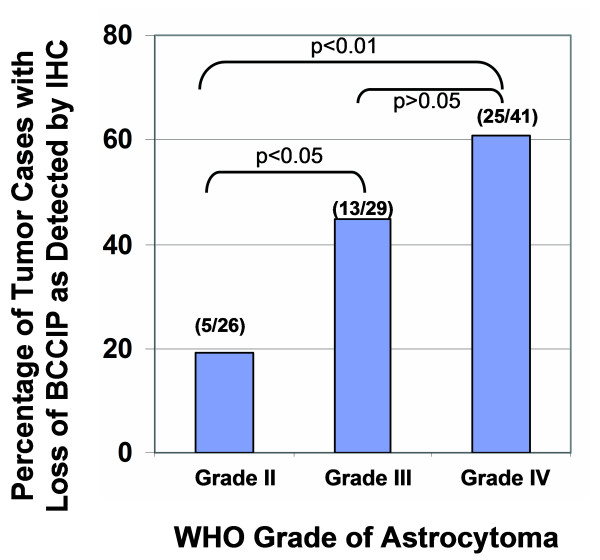
**Frequency of loss of BCCIP protein expression in different WHO grades of astrocytomas **[30]. Shown is the percentage of astrocytomas that are BCCIP negative. The p-values indicate the statistic values between the indicated tumor grade groups.

Then, we stained serial sections of the tumor tissue arrays with anti-GFAP and anti-BCCIP antibodies. Because BCCIP is mainly expressed in GFAP positive cells, we grouped the tumors based on their GFAP status (Table 1). As shown in Table 1, among 91 cases of GFAP positive astrocytomas, 40 were BCCIP negative, an overall rate of 44.8%. Three of the five GFAP negative astrocytomas are also BCCIP negative. It has been established that alteration of 10q25.3–26.1 is associated with high grade and poor prognosis of astrocytic tumors [7-9,36]. Interestingly, we found that 5 out of 26 cases (19.2%) WHO grade II astrocytomas are BCCIP negative, whereas 13/29 (44.8%) WHO grade III and 25/41 (60.1%) WHO grade IV astrocytomas are BCCIP negative (Figure 3). Based on a *Chi-*square test, there is a statistically higher frequency of BCCIP negativity in grade III (p < 0.05) and IV (p < 0.01) tumors than that of the grade II tumors, but there was no statistic difference between grades III and IV (p > 0.05). The correlation of lacking BCCIP expression with the WHO grade is consistent with the association between 10q26 abnormalities and the poor prognosis of astrocytomas [7-9,36]. In addition, 2 out 6 GFAP positive oligodendroglioma are BCCIP negative, and all 5 cases of ependymomas are GFAP negative but BCCIP positive.

**Table 1:** Number of cases analyzed by IHC on tissue array

	GFAP (+)			GFAP (-)		
	BCCIP (+)	BCCIP (-)	Total	BCCIP (+)	BCCIP (-)	Total
Astrocytic tumors						
Astrocytoma (Grade II)	21	5	26			
Anaplastic Astrocytoma (Grade III)	14	12	26	2	1	3
Glioblstoma (Grade IV)	16	23	39	0	2	2
Total	51	40	91	2	3	5

Oligodendroglioma						
Oligodendroglioma (Grade II)	1	1	2			
Anaplastic Oligodendroglioma (Grade III)	3	1	4			
Total	4	2	6			

Ependymoma				5	0	5
Total				5	0	5

## Discussion

Cytogenetic and LOH analyses have suggested that at least three distinct regions of the long arm of chromosome 10 are related to brain tumorigenesis: 10q23.3, 10q24–25, and 10q25.3–26.2. The tumor susceptibility gene located at 10q23.3 has been identified as the PTEN (phosphatase and tension homologue detected on chromosome TEN) or MMAC (mutated in multiple advanced cancers) gene. PTEN/MMAC protein has two distinct structural domains, the phosphatase domain and a C2 domain that binds phospholipid membranes in vitro [37]. PTEN mutations have been identified in a variety of human cancers [38-41]. A second tumor susceptibility gene is located at 10q24–25. The c-Myc, Max homologous gene MXI1 is located in this region [17,42-50].

A putative tumor susceptibility gene DMBT1 (Deleted in Malignant Brain Tumors) has been identified in 10q25.3–26.2. [51]. DMBT1 is homologous to the scavenger receptor cysteine-rich (SRCR) superfamily of proteins. Since it is deleted in a large proportion of brain tumors, it has been suggested as a candidate for the tumor susceptibility gene in this region. However, expression of DMBT1 in tumor cells has little effect on tumor cell growth or tumor associated phenotypes [4,52-54]. This opens the possibility that additional genes in this region may be relevant to brain tumorigenesis. Considering that: 1) BCCIP interacts with BRCA2 and p21, 2) BCCIP plays a role in homologous recombination, cell cycle regulation, p53 transcription activity, and chromosome instability [19-24,26,27], 3) BCCIP expression is absent in a significant portion of astrocytomas (Figure 2), and 4) the lack of BCCIP expression is correlated with the aggressiveness of astrocytomas (Figure 3), we suggest that BCCIP is a strong candidate for the tumor suppressor gene in the chromosome 10q26 region. However, additional investigation, preferably with animal knock-out or knock-down models, is needed to conclude whether BCCIP defects directly cause brain tumor. It should be pointed out that the 10q26.2 region has been implicated in many other cancer types. The role of BCCIP in other cancer types warrants additional investigations.

Mutation of p53 has been reported in 31% of brain tumor, but LOH of the 10q region has been reported in 69% of brain tumor [9]. Despite that majority of the brain tumor harbors wild type p53, brain tumors are well known for their radiation resistance. We reported that in the absence of BCCIP, the transactivation activity of wild type p53 protein is not sustainable [26]. Down-regulation of BCCIP leads to a resistance to ionizing radiation of HCT116 cells with wild type p53 [26]. It has been recently shown that lack of BCCIP in p53 wild type laryngeal cancer is associated with poor progress in response to radiation therapy [55]. Here we show that a significant portion of astrocytomas has down-regulation of BCCIP. It would be interesting to determine whether the lack of BCCIP expression plays a role in the radiation resistance for these brain tumors with wild type p53.

The dependence of p53 function on BCCIP makes an ideal argument that BCCIP may function as a tumor suppressor, and BCCIP defects are responsible for astrocytoma aggression and resistant to radiation therapy. Because BCCIP has also been shown to be required for completion of mitosis, and severe knockdown of BCCIP resulted in growth retardation in other cell types [27], it remains to be addressed how loss of BCCIP expression may lead to brain tumor survival. Thus, it is likely that lack of BCCIP expression needs to be coupled with additional genetic alterations for tumor cells to be viable and remain aggressive. If this is true, then identification of these additional genetic factor(s) enabling BCCIP defective cells to survive is a critical issue for future studies.

## Conclusion

We found that BCCIP expression is down-regulated in a significant portion of astrocytomas, and the loss of BCCIP expression is more frequent in the aggressive form of astrocytomas. These data suggest BCCIP as a potential marker for brain tumorigenesis.

## Competing interests

The authors declare that they have no competing interests.

## Authors' contributions

JL designed and conducted the quantitative PCR analysis, and drafted a portion of the manuscript. HL conducted the IHC experiments and drafted a portion of the manuscript. ZS designed the study, performed data analysis, and finalized the manuscript, HO provided 1/3 of the DNA samples and evaluated some of the IHC slides, AM provided the 2/3 of tumor DNA samples. Both HO and AM provided inputs on improving an early draft of the manuscript.

## Pre-publication history

The pre-publication history for this paper can be accessed here:

http://www.biomedcentral.com/1471-2407/9/268/prepub
